# Dual-modality fluorescence lifetime imaging-optical coherence tomography intravascular catheter system with freeform catheter optics

**DOI:** 10.1117/1.JBO.27.7.076005

**Published:** 2022-07-21

**Authors:** Cai Li, Julien Bec, Xiangnan Zhou, Laura Marcu

**Affiliations:** University of California, Department of Biomedical Engineering, Davis, California, United States

**Keywords:** intravascular imaging, catheter, fluorescence lifetime, optical coherence tomography, atherosclerosis

## Abstract

**Significance:**

Intravascular imaging is key to investigations into atherosclerotic plaque pathobiology and cardiovascular diagnostics overall. The development of multimodal imaging devices compatible with intracoronary applications has the potential to address limitations of currently available single-modality systems.

**Aim:**

We designed and characterized a robust, high performance multimodal imaging system that combines optical coherence tomography (OCT) and multispectral fluorescence lifetime imaging (FLIm) for intraluminal simultaneous assessment of structural and biochemical properties of coronary arteries.

**Approach:**

Several shortcomings of existing FLIm-OCT catheter systems are addressed by adopting key features, namely (1) a custom fiber optic rotary joint based on an air bearing, (2) a broadband catheter using a freeform reflective optics, and (3) integrated solid-state FLIm detectors. Improvements are quantified using a combination of experimental characterization and simulations.

**Results:**

Excellent UV and IR coupling efficiencies and stability (IR: 75.7%±0.4%, UV: 45.7%±0.35%) are achieved; high FLIm optical performance is obtained (UV beam FWHM: 50  μm) contemporaneously with excellent OCT beam quality (IR beam FWHM: 17  μm). High-quality FLIm OCT image of a human coronary artery specimen was acquired.

**Conclusion:**

The ability of this intravascular imaging system to provide comprehensive structural and biochemical properties will be valuable to further our understanding of plaque pathophysiology and improve cardiovascular diagnostics.

## 1 Introduction

Cardiovascular disease (CVD) is currently the leading cause of death globally.^1^ Early detection and improved characterization of atherosclerosis is necessary for the preventive care of CVD to protect patients from the clinical manifestation such as myocardial infarction, stroke, and peripheral artery disease. Therefore, how to effectively detect and characterize the nature of the atherosclerotic plaques has become a major topic in CVD studies and has driven the development of a variety of cardiovascular imaging technologies.^2^

Although some clinical imaging techniques like cardiovascular angiography (CA) can provide the two-dimensional image of lumens, they are limited by the resolutions and lack the capability of analyzing the atherosclerosis plaque characteristics.^3^ In recent years, many intravascular imaging techniques have been developed. For example, intravascular ultrasound (IVUS) has been widely used in clinics to image the lumens with decent resolution and penetration depth.^4^ Intravascular optical coherence tomography (IV OCT) can generate high-resolution structural images at the expense of penetration depth and has been proven valuable tool for cardiovascular diagnostics by providing accurate vessel sizing, identify the presence of atherosclerosis lesions and some of their key features (e.g., extracellular matrix, macrophages, and cholesterol crystals) and assess stent apposition.^5^ However, neither of these two techniques are sensitive to change in tissue biochemical property, making it difficult to characterize the plaque composition, a key determinant for plaque behavior and subsequent cardiovascular events.^6^ Given this limitation, new molecular imaging techniques capable of complementing IVUS or IV-OCT are intensively researched.^7^^,^^8^

Fluorescence lifetime imaging (FLIm) measures the fluorescence decay characteristics of fluorescent species in tissues and cells. FLIm can be used to identify endogenous fluorophores which may not be identified using spectral properties alone.^9^ The application of FLIm to the characterization of CVD by identifying plaque components such as collagen, elastin, and macrophage foam cells using both benchtop^10^^,^^11^ and intravascular imaging systems^12^ has been reported. In addition to the increased specificity compared to steady-state fluorescence, another advantage of FLIm is that the average fluorescence lifetime is an intrinsic property of tissue that reflects the relative abundance of fluorophores. Variations in signal intensity due to variations of excitation/ collection geometry during helical scans lead to variations in signal-to-noise ratio (SNR) of the FLIm signal which in turns influences the measured lifetime variability, but the expected lifetime value is not affected. By contrast, distance-based intensity correction is required for intravascular intensity-based fluorescence^13^ and accurate estimation of the fluorescence is further complicated by the influence of tissue absorption and scattering properties.^14^ Our group has combined FLIm with IVUS and demonstrated FLIm’s capability of characterizing atherosclerosis in coronary arteries.^8^^,^^12^ However, intravascular FLIm requires flushing and a fast pullback, for which the speed of conventional IVUS is a limiting factor. Additionally, the integration of both ultrasound and optical channels increases the device’s complexity and limits the potential for miniaturization. More recently, single-fiber FLIm and OCT has been demonstrated both for raster scanning^15^ and intraluminal^16^^,^^17^ applications and addresses some of the limitations of FLIm and IVUS integration. Nevertheless, the performance of previously reported implementations of intraluminal FLIm-OCT is limited by some design tradeoffs stemming from the multimodal integration.

In this paper, we will present an intravascular OCT and FLIm imaging system that addresses some of the limitations of previously reported implementations,^16^^,^^17^ in terms of catheter optics, motor drive design, and FLIm integration. We first describe and characterize key novel aspects of the a new bimodal FLIm-OCT apparatus consisting of (1) a catheter combining a DCF with a broadband reflective distal end optic,^18^ (2) a high-efficiency air-bearing fiber optic rotary coupler, and (3) solid state detectors integrated into the motor drive unit (MDU) to perform multispectral FLIm data acquisition. Then, we report simulation results of the catheter performance in comparison with previously reported ball-lens FLIm-OCT catheter. Finally, we demonstrate the system’s ability to acquire coregistered biochemical and structural information from diseased human artery and corroborate these findings with histological findings.

## 2 Methods and Materials

### 2.1 FLIm-OCT System Description

**Fig. 1:**
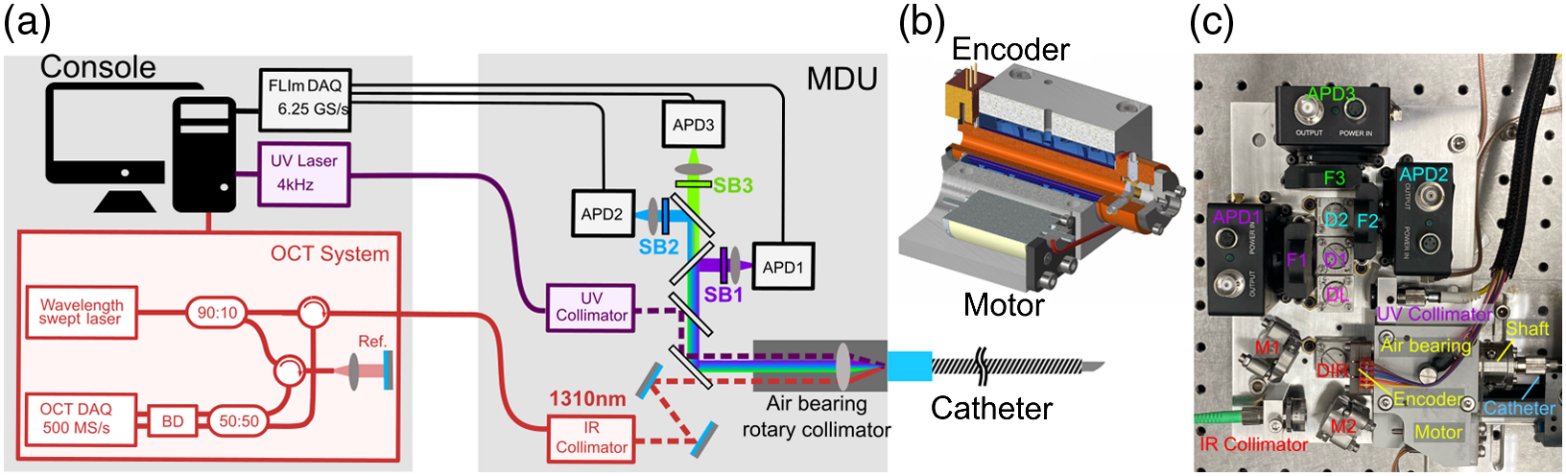
(a) FLIm-OCT system schematic. The system is composed of an imaging catheter, an MDU, FLIm, and OCT modules. (b) 3D model of rotary collimator. The motor drives the rotation of shaft. The encoder read out of the angular position of the rotating shaft is used for closed-loop control of the shaft rotation as well as image reconstruction. (c) Top view of the MDU highlighting the OCT path, FLIm excitation and detection paths, and rotary collimator.

The dual-modality system is composed of an imaging catheter, an MDU, and a console as depicted in Fig. 1. It includes OCT and FLIm-specific components in the console and MDU, as well as shared components that enable bimodal imaging, distributed between catheter and MDU, as described in the following.

### 2.2 FLIm Module

The FLIm capability relies on a pulse sampling implementation, derived from instrumentation previously developed for multimodal intravascular use,^19^ and that incorporates individual solid-state detectors, similar to a FLIm instrument designed for intraoperative use recently developed by our group.^20^ The excitation light source consists of a pulsed 355-nm diode-pumped solid-state laser (4-kHz repetition rate, 2-μJ pulse energy, STV-02E-140, TEEM photonics, France). The excitation light is first transmitted to the MDU with a 50-μm core multimode fiber (Polymicro FVP050055065, Arizona, United States) and then coupled into the catheter as described in Sec. 2.5. The FLIm signal detection is implemented by first resolving the fluorescence signal into three spectral bands (390/40, 450/45, and 540/50  nm) by a set of dichroic mirrors and bandpass filters.^19^ The optical signal from each spectral band is then focused (C610TME-A, Thorlabs, New Jersey, United States) into the sensitive area of individual photodetector modules (APD430A2-SP1, Thorlabs, New Jersey, United States). Each module consists of a UV-enhanced avalanche photodiode (APD) (200-μm diameter sensitive area, S12053-02, Hamamatsu, Japan), adjustable biasing circuitry, and trans-impedance amplifier (5  kV/A). The output from each APD module is sampled by a 6.25  GS/s digitizer (NI PXIe-5185, National Instruments, Austin, Texas, United States).

### 2.3 OCT Module

The intensity-based swept-source OCT (SS-OCT) is implemented as described in our previous work based on an OCT OEM engine from Axsun Technology.^15^ It includes a 1310±55  nm swept-source laser and enables an A-scan rate of 50 kHz. The interferometry is realized with fiber couplers for splitting the laser output from the OEM engine to the sample arm and reference arm and mixing the reflected light from both arms. A single-mode fiber (SMF) is used in the reference arm to reduce the ghost artifacts caused by the light leakage to the multimode cladding, different from previous work.^15^ The material of the SMF core is the same as that of DCF to avoid introducing dispersion. The sample arm light is coupled from the OCT modality to the MDU by an SMF and finally coupled to the DCF based fiber probe. The interference signal is generated in the fiber coupler and a 180-deg phase shift is introduced between the two outputs of the fiber coupler.^21^ The differential signal of these two outputs is detected with the dual balanced detector and sampled by a 12 bit, 500  MS/s FPGA DAQ board in the OCT engine.

### 2.4 Motor Drive Unit

The MDU fulfills multiple roles of mechanical actuation of the catheter, multiplexing and demultiplexing of FLIm and OCT excitation and collection light, and detection of the FLIm signal. A UV collimator and an infrared (IR) collimator are used to generate collimated UV (FLIm) and IR (OCT) beams. The UV beam is reflected by a first dichroic mirror used to separate FLIm excitation and fluorescence emission light (Di01-R355, Semrock, Rochester, NY), and then merged with the OCT beam by a second dichroic mirror (FF735-Di02-25x36, Semrock, Rochester, New Jersey, United States). A periscope was integrated within the OCT optical path provide the four degrees of freedom required for proper beam alignment. The overlapping IR and UV beams are sent to a custom fiber optic rotary collimator (FORC) (Fig. 1).

The FORC is based on a stationary air bearing (OAV0500IB, Princeton, New Jersey, United States) and a rotating shaft. The shaft comports an FC/PC fiber receptacle (for connection with the catheter’s imaging core) as well as a focusing lens (11-mm focal length, A397TM, Thorlabs, New Jersey, United States) adjustable over three degrees of freedom (two orthogonal continuous lateral adjustments and discrete axial adjustment) that enables focusing of the IR and UV beams into the catheter’s core and inner cladding, respectively (see Sec. 2.5). The FORC shaft is driven by a brushless motor (EC-max 22, Maxon motors, Sachseln, Switzerland). Angular position information of the shaft used for FLIm and OCT image reconstruction is provided by the optical angular encoder (US Digital, Vancouver, Washington, United States) and used as the feedback to the shaft rotation speed controller (ESCON 24/2, Maxon Motors, Sachseln, Switzerland) [Fig. 1(b)].

Both fluorescence emission and OCT backscattered signal transmitted by the catheter are collimated back by the FORC lens. The IR OCT beam is transmitted to the IR collimator for OCT detection, whereas the fluorescence optical signal is spectrally resolved into three different spectral bands and directed to the respective APD modules (see methods Sec. 2.2).

### 2.5 Rotational Dual-Modality Catheter

The catheter consists of a rotating imaging core housed in a saline solution-filled sheath. The 1.6-m imaging core consists of a double-clad fiber (SM-9/105/125-20A, Nufern, East Granby, Connecticut, United States) enclosed in a two-layer torque coil (Asahi Intecc, Aishi, Japan). The proximal end of the imaging core was terminated with a 150-mm stainless steel shaft and an FC/PC connector for connection to the FORC shaft. A broadband freeform reflective micro-optic was integrated at the distal end of the imaging core; beam reflection off the curved aluminum-coated reflective surface enables both beam redirection and accurate focusing over an extended wavelength range.^18^ The DCF and the freeform optic were actively aligned by coupling 1310-nm light into the fiber’s core and adjusting the fiber/s relative position in the vertical direction to obtain the nominal beam forward tilt. The optic was then attached to the fiber using UV-curing acrylate optical adhesive (OG603, Epotek, Billerica, Massachusetts, United States). The imaging sheath is composed of a 4-Fr braided PEBAX shaft (Merit Medical, Utah, United States), terminated with an optically transparent polymethylpentene 1.25-mm outer diameter imaging section (Vesta Inc., California, United States).

### 2.6 Data Acquisition and Processing Methods

Data was acquired using a catheter rotation speed of 10 rps to enable sufficient FLIm angular sampling with the current laser (4 kHz, 400 points/rotation). The average fluorescence lifetime for each spectral band was processed for each point measurement using a Laguerre basis expansion technique.^22^ For OCT processing, the A-lines were obtained by Fourier transforming the measured spectra. To suppress the fixed pattern noise observed in the signal, the reference spectrum extraction was applied.^23^ Cross-sectional OCT images (B-scans) were reconstructed based on the angular information obtained from the FORC encoder. Finally, lifetime color-coded B-scans for each FLIm spectral band were generated by interpolating the average lifetime values for each OCT A-line angular location and setting each A-line’s color based on the color-coded average lifetime value.

### 2.7 Fiber Optic Rotary Collimator Alignment and Characterization

A key figure of merit of the FORC performance is the coupling efficiency (CE) of light between the FLIm OCT modality and the catheter (355 to 1365 nm). A high and stable CE shall be achieved during the rotation of the FORC shaft. Light coupling between the core of the OCT subsystem fiber and the core of the rotating imaging DCF is more challenging due to the small core sizes (9  μm) than the coupling of FLIm optical beams to and from the DCF’s inner cladding (105-μm diameter); therefore, we optimized the alignment of FORC by maximizing the OCT coupling performance.

**Fig. 2:**
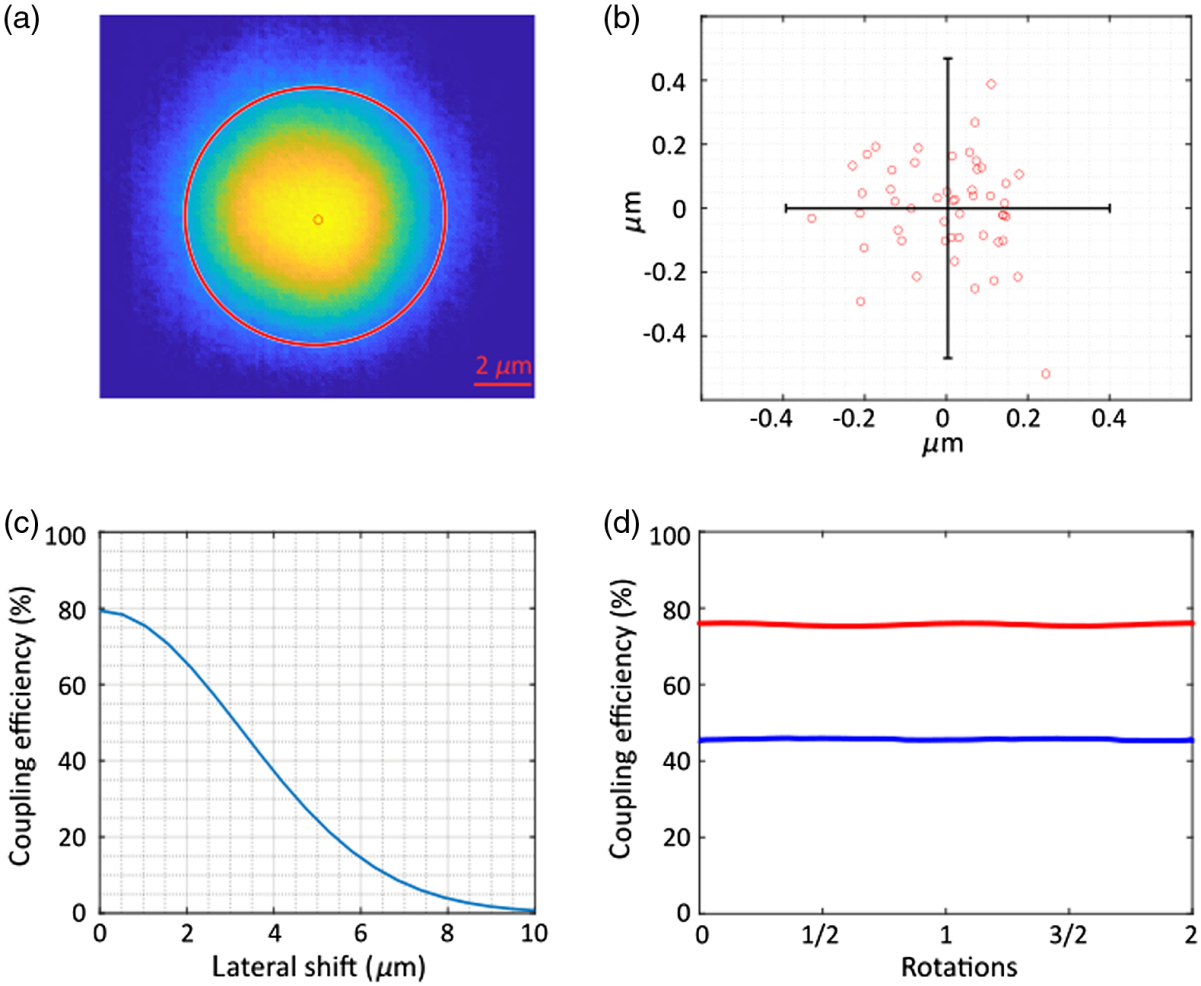
(a) Beam profile of the focused IR beam after the OCT IR collimator lens. The large red circle has a diameter of 9  μm, same as the fiber core. The small red circular spot is the calculated beam centroid. (b) Trace of beam centroid positions when rotating the FORC. (c) The simulated single-mode CE over the later shift of beam spot relative to the fiber core center. (d) The measured IR and UV CE during the rotation of FORC.

The IR CE was maximized by first minimizing the eccentricity of the collimated single-mode beam exiting the FORC, measured from the image of the focused IR spot behind the OCT collimator lens [Fig. 2(a)]. The measurement was performed using a custom beam profiler based on a camera (½″ CMOS sensor, 3.1MPix, AmScope, Irvine, California, United States), 100× Plan Apo objective, and a 150-mm focal length tube lens that provided a pixel size of 0.08 mm. Variation of the beam’s centroid location was minimized by adjusting the lateral position of the FORC lens during the shaft rotation [Fig. 2(b)]. In a second step, optical coupling of the rotary fiber to the stationary fiber was maximized by adjusting the vertical/horizontal position and yaw/pitch of the IR beam directed to the OCT collimator by adjusting the two flat mirrors (M1, M2), as well as the IR collimator focus. The effect of IR beam misalignment was investigated by calculating the CE of single-mode coupling versus the offset between beam centroid and fiber core center using physics optics propagation module in Zemax [Fig. 2(c)].

The UV excitation coupling was then maximized by adjusting the two dichroic mirrors on the UV path [Fig. 1(b): DIR, DL] as well as the focusing of the UV collimator.

Finally, the coupling efficiencies of OCT IR (1310  nm±55  nm) and FLIm UV excitation light (355 nm) were acquired by measuring the power before and after the FORC [Fig. 2(d)].

### 2.8 Rotational Catheter Performance Characterization

**Fig. 3:**
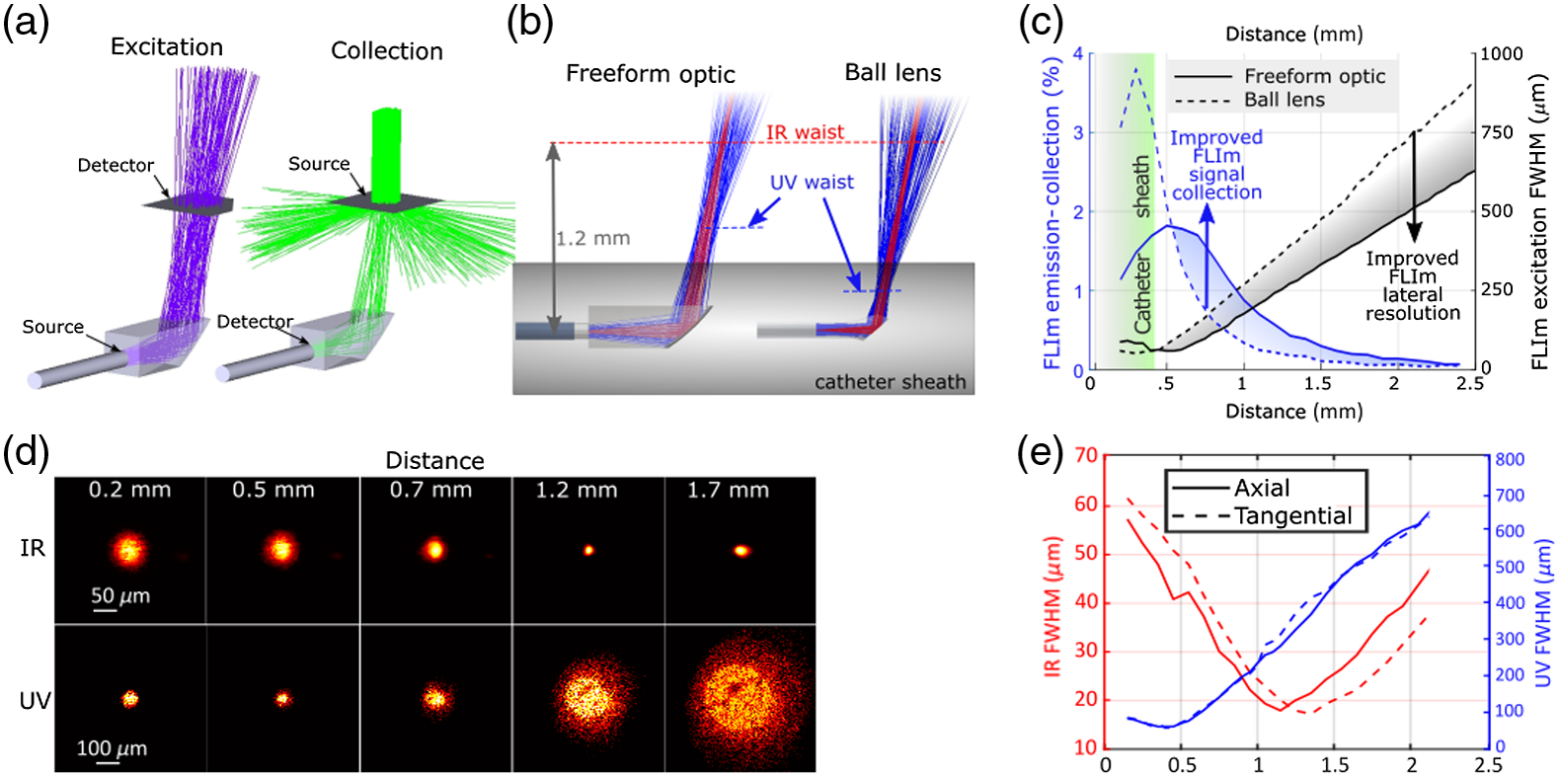
(a) Ray-tracing model for simulating the FLIm excitation and emission collection. (b) Ray tracing comparison between the freeform optics and ball lens probes. (c) Comparison of FLIm excitation FWHM and emission collection efficiency between the freeform optic and ball lens probes. (d) IR and UV beam profiles at different distances from the optical axis. (e) The UV and IR FWHM were calculated from the beam profiles versus the distance from the optical axis.

The FLIm lateral resolution and collection efficiency of the freeform reflective optic were compared using a ray-tracing simulation of the performance obtained from a side-viewing ball lens described in a recent multimodal catheter publication.^17^ A nonsequential model was built using the simulation model of the freeform reflective optic and the dimensions of the side-viewing ball lens probe [Fig. 3(a)]. A 105-μm diameter circular source was used to simulate the light exiting the DCF cladding with the divergence computed from the NA of the inner cladding. For simplicity, the glass tube surrounding the ball-lens design was omitted. The axial and lateral resolutions were obtained by simulating the beam profile and computing the FWHM in both directions at multiple distances from the optical axis. The beam centroid positions were also computed and used to simulate each design’s collection efficiency with respect to target distance. For each distance from the fiber’s optical axis, a circular Lambertian source whose diameter equals the RMS diameter of the excitation beam obtained at the previous step was defined by illuminating a collimated beam to the Lambertian scattering plane. A circular detector with an acceptance angle defined from the fiber’s inner cladding NA and located in correspondence to the fiber’s distal end was used to compute the collection efficiency, defined as the ratio of detected photons and emitted photons.

The FLIm excitation beam (355 nm) and OCT IR beam (1310 nm) were measured at different distances from the fiber’s optical axis using a second custom beam profiler based on a camera (½″ CMOS sensor, 3.1 MPix, AmScope, Irvine, California, United States), 20× Plan Apo objective and a 40-mm focal length tube lens that provide a pixel size of 1.4  μm.

### 2.9 Human Coronary Artery Imaging

The artery specimen was provided by the University of Pennsylvania heart transplant program in accordance with current legal requirements and guidelines and was approved by the University of Pennsylvania Hospital Institutional Review Board as well as UC Davis Biological Use Authorization. This 50-mm long coronary artery section was immersed in phosphate-buffered saline, a guidewire was inserted in the segment, the FLIm-OCT catheter was inserted, and the guidewire was removed before imaging to obtain a full view of the vessel without guidewire shadow. After imaging, it was inked, fixed (10% neutral-buffer formalin), sectioned in ∼2.5-mm-long rings, and embedded in paraffin. Sections (5-μm thick) were stained with hematoxylin and eosin stain and modified Movat’s pentachrome. Anti-CD68 antibodies were used to identify macrophages. Registration of imaging data with histology was performed based on angular registration of the container bottom surface (visible at the beginning of the pullback) and the top surface inking of the artery sample. Axial registration was performed based on pullback location and ring number. Final adjustments of the registration were performed by matching morphological features between the OCT data and histological sections.

## 3 Results

### 3.1 FORC Performance Evaluation Results

The stability of free-space beam characteristics (position and dimensions) during rotation is key to ensure efficient single-mode fiber coupling. Figure 2(a) shows the focused IR beam after the OCT collimator lens and the red circle represents the fiber core with a diameter of 9  μm. That demonstrates a good match between the sizes of fiber core and focused beam profile. The beam centroid was calculated from the beam profile [Fig. 2(a)] and traced when rotating the FORC. Figure 2(b) depicts the beam centroid positions when rotating the FORC, after the alignment was optimized. The residual eccentricity of the optical axis of the FORC during the rotation generates a shift of the beam centroid position with respect to the location of the OCT stationary collimator fiber core. This shift, characterized by three times the standard deviation of the centroid coordinates, was measured to be 0.40 and 0.47  μm in the lateral and vertical directions, respectively. Figure 2(c) shows the simulated CE when the beam centroid is laterally offset from the fiber core center. For a 0.5-μm offset, an expected coupling of 78% was obtained. Figure 2(d) shows the measured IR and UV CE for the OCT and FLIm excitation during the rotation of FORC. The average IR CE was 75.7%, which is very close to the simulation result. The min-max difference was 0.8%, indicating an extremely stable coupling to the fiber core. The average UV CE is 45.7%, with a min-max difference of 0.7% demonstrating high stability.

### 3.2 Freeform Reflective Optics Evaluation

The optical performance of freeform optic and ball lens was evaluated from raytracing simulation as described in Sec. 2.8 and compared as shown in Figs. 3(b) and 3(c). Figure 3(b) shows that the waist of the UV beam obtained from the ball lens catheter is located inside the catheter sheath (2.6-Fr diameter), thus all the FLIm measurements are performed with the vessel wall located past the beam waist, reducing both lateral resolution and collection efficiency. The freeform optic significantly improves the UV waist position, now located at ∼100  μm from the sheath’s outer surface, and the excitation beam divergence is also drastically reduced. This improvement resulted in an improved FLIm lateral resolution as shown by the FLIm excitation beam full width at half maximum [FWHM, Fig. 3(c), black curves]. The excitation beam FWHM using the freeform optics is ∼30% smaller than that using the ball lens. The emission collection efficiency is also improved significantly by using the freeform optic for all distances above 0.6 mm from the catheter’s central axis [Fig. 3(c), blue curves]. Variations in signal collection due to variations of catheter to vessel wall distance at different angular positions of the core are detrimental to FLIm signal SNR. This variation, expressed as ratio of FLIm emission/collection efficiency at the catheter’s surface and 1.5-mm distance is much lower for the freeform optic (R=5.6) than the ball lens design (R=29.1), demonstrating another key benefit of this implementation.

The performance of freeform optic was validated by beam profiling. The beam profiles obtained for both IR (delivered through the fiber core) and UV (delivered through the inner cladding) are displayed in Figs. 3(d) and 3(e). For IR and UV, beam waist FWHM of 17 and 50  μm, respectively, are obtained. The position of the beam waist is also in good agreement with ray-tracing simulations [Fig. 3(b)]. The OCT IR beam is focused at 1.15 to 1.35 mm from the probe axis with a small astigmatism (0.2-mm focus offset between the beam in tangential and axial directions). The FLIm UV beam is focused at 0.4 mm from the probe axis without astigmatism.

### 3.3 Artery Results

**Fig. 4:**
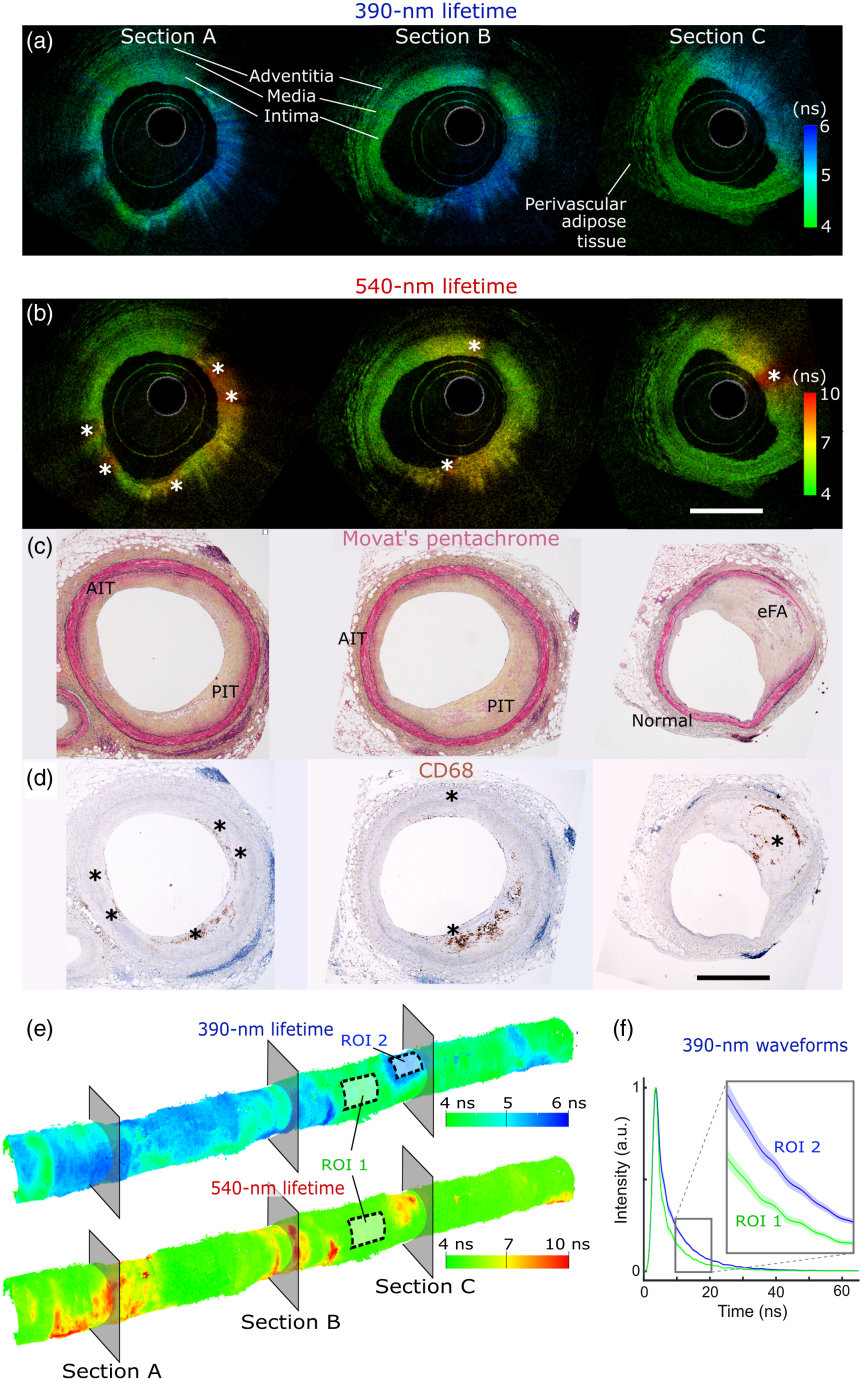
Imaging of human coronary specimen. FLIm/OCT B-scans where brightness corresponds to the OCT backscattered intensity whereas the Hue corresponds to the FLIm averaged lifetime for both (a) the 390-nm spectral band and (b) 540-nm spectral band. The OCT penetration depth enables the visualization of the full vessel wall in healthy regions, including some perivascular adipocytes. Movat’s pentachrome sections highlight that low 390-nm lifetime is associated with healthy regions (Normal, AIT), whereas lifetime increase is associated with PIT and early FA (eFA) regions (c). (d) CD68-stained sections highlight that increase in 540-nm lifetime corresponds to areas of superficial foam cells accumulation. 3D color-coded volumetric images enable the identification of high FLIm-contrast areas over the entire vessel segment. (e) The location of each B-scan (A/B/C) is represented by the cross-sectional planes. FLIm measurement variability was estimated by computing average lifetime mean and standard deviations for two homogeneous ROIs. ROI 1 (390-nm band): 3.82/0.15 ns. ROI 1 (540-nm band): 4.86/0.14 ns. ROI 2 (390-nm band): 5.21/0.13 ns. Mean FLIm waveforms (curve) and standard deviation (shaded area) from ROIs 1 and 2 demonstrate clear differences and high SNR (f) (Video 1, mp4, 6.15 MB [URL: https://doi.org/10.1117/1.JBO.27.7.076005.1]).

Three sections of color-coded FLIm-OCT renderings are displayed in Fig. 4 with matching Movat’s pentachrome and CD68 stained sections. First, the penetration depth of OCT enables visualization of the whole vessel wall with well-resolved intima/media/adventitia layers as well as perivascular adipose tissue visible in normal and adaptive intimal thickening (AIT) regions [Figs. 4(a) and 4(b)].

As expected, OCT attenuation is higher in pathological intimal thickening (PIT) and fibroatheroma (FA) regions. 390-nm lifetime contrast shows consistent low values in healthy and AIT regions, but increased lifetime values in PIT and FA regions, as expected based on earlier work^12^ that associated increased 390-nm lifetime increase with presence of recently formed matrix (collagen, proteoglycans) often present in PIT and FA regions.

The vessel segment imaged here presents few locations of dense macrophage foam cells (mFC) accumulation in the vicinity of the lumen (<150  μm, expected penetration depth of FLIm), but locations of superficial mFC accumulation are well-matched with locations of increased 540-nm lifetime. Based on these observations, the FLIm spatial resolution is sufficient to obtain strong contrast even in presence of small mFC clusters (Section C, <100-μm wide). Conversely, no mFC could be identified in areas of low 540-nm lifetime, highlighting the specificity of the 540-nm contrast with respect to mFC detection. Finally, 3D renderings of FLIm-coded OCT [Fig. 4(e)] facilitate the identification of possible disease location over the whole artery segment. Clear separation between FLIm waveforms obtained from regions of interests (ROIs) 1 to 2 [Fig. 4(f)] illustrate the high SNR of the proposed direct-detection solid-state pulse-sampling approach, obtained without application of any spatial filtering. This high SNR translates into distinct lifetime values obtained in the 390-nm spectral bands in both normal regions (region 1, 3.82/0.15 ns mean/standard deviation) and lesion surfaces (region 2, 5.21/0.13 ns mean/standard deviation).

## 4 Discussion

The successful design and implementation of an FLIm-OCT catheter system should maximize optical throughput for each modality while achieving stable and reliable operation. These guiding principles are reflected in the design of the rotary junction, integration of the FLIm detection into the MDU, and in the bimodal catheter design as discussed in the following section.

The optical rotary junction, composed of the FORC and the stationary UV and IR collimators is a key component of the system. Lensed applications where a set of two stationary/rotating lenses are integrated with the optical path are well suited for single wavelength or narrow wavelength ranges, but adaptation of these designs to accommodate the wide wavelength range required for some multimodal applications (355 to 1360 nm here) is hindered by chromatic focal shift. Lensless rotary junctions have been developed as an alternative^24^ but the requirement of index matching fluid and the contact between rotary fiber, stationary fiber, and guiding ferrule makes their long-term stability and reliability unclear. A more promising approach consists of a free-space lensed design with increased separation between rotary and stationary elements, such that a dichroic integrated within the optical path enables the use of separate collimating lenses for OCT/ FLIm excitation and FLIm collection, thus enabling some compensation of the chromatic focal shift of the common rotating lens.^16^

Stable, high transmission coupling of such a lensed rotary junction requires the minimization of angular and radial runout of the shaft in order to achieve low pointing error.^25^ Most designs are based on duplex ball bearings,^26^ for which minimizing radial runout requires high-quality bearing and tight tolerances in the machining of the shaft and bearing housing bore. Additionally, ball-bearings tend to present a runout error with large contributions from higher harmonics^27^ which cannot be corrected by adjusting the position of the collimator lens. In contrast, air bearings present low total indicated runout and high radial stiffness (<±0.025  μm, >10  N/mm). These characteristics, in addition to low friction, high-speed operation, absence of vibrations, and relaxed requirements on mechanical tolerances make air bearing ideally suited for the manufacturing of high-performance, cost-effective free space optical rotary junctions. Another key feature of the rotary junction is the integration of the drive motor.

Coupling of the motor to the shaft with a timing belt generates vibrations from the meshing of the belt teeth with the pulley but circular belts may slip and cause issues in setting an accurate rotation speed or determine the exact angular position of the shaft based on information from the motor. This limitation was addressed by implementing closed-loop speed control feedback to the motor from the angular encoder on the main shaft to achieve both accurate rotation and smooth operation and provide positional information for image reconstruction.

The implementation reported here also differs from previously reported FLIm-OCT catheter systems^16^^,^^17^ by integrating individual FLIm solid-state detectors in the MDU to avoid the coupling losses caused by the additional transmission fiber required when FLIm detection is performed in the system’s cart. Direct free-space coupling of the fluorescence signal into individual detectors facilitates the correction of the chromatic focal shift for each of the detection bands independently. These detectors also present increased quantum efficiency leading to increased FLIm signal quality when compared to implementations using microchannel plate photomultiplier tubes.^20^

The performance of the multimodal system is also determined by the ability of the catheter to support both OCT and FLIm imaging with minimal trade-offs. The efficient use of DCF for multimodal OCT applications has been extensively documented as is the transmission of 355-nm excitation light through the inner cladding.^16^ On the contrary, the use of side-viewing ball lens probes derived from single-modality OCT, where excitation light is transmitted in the inner cladding, leads to poor spatial resolution due to internal reflection of the excitation light before reaching the ball lens itself.^16^ Improvements in lateral resolution can be addressed by using longer 375-nm excitation light in the DCF’s core^17^ at the cost of solarization issues that limit the amount of core-transmitted excitation light. The low aperture of the ball lens design, in combination with manufacturing constraints, limits both collection efficiency and working distance. On the other hand, the results presented here (Fig. 3) demonstrate that the combination of a DCF with a freeform reflective optic is ideally suited for intravascular FLIm.^18^ Beam profiling results reported for OCT are consistent with previously reported values,^18^ demonstrate an improvement compared to the 25-mm FWHM typical of OCT catheters currently used in clinics^28^ and are similar to new designs recently reported for intravascular OCT once accounting for differences in working distance.^29^ It is worth mentioning that the freeform optic design used for this application was only optimized for FLIm and further improvements in OCT performance may be achieved by further optimization.

Optical losses are further reduced by simplifying the interface between the catheter and the rotary junction compared with previously reported systems.^16^^,^^17^ Both of these earlier designs include a short fiber section on the rotary side of the joint and require physical contact with the catheter’s proximal connector.^16^^,^^24^ Physical contact is widely used for single-mode applications where direct contact between cores enables low insertion losses (typ. <0.2  dB) but multimode transmission through the inner cladding suffers from the presence of two additional air/glass interfaces, which are avoided by coupling the catheter connector directly into the rotary junction as reported here.

With the pulse sampling approach used to acquire FLIm data, FLIm average lifetimes are obtained from each individual UV laser pulse. Currently, the system’s framerate of 10 fps (frames per second) is constrained by the targeted number of FLIm points per rotation (n=400) and the repetition rate of the laser used for this demonstration (4 kHz). For future work, the adoption of a 30-kHz repetition rate 355-nm laser (e.g. SNV-60P-10x, TEEM photonics, France) will enable operation at 100 fps with a small reduction of the number of FLIm points per rotation (n=300). In this configuration, the framerate will match currently available clinical intravascular OCT systems. Additionally, the 1.25-mm outer diameter of the imaging section originates from the size of the available extrusion, carried over from earlier designs. It will be reduced to closely match the imaging core’s outer diameter and achieve dimensions similar to current clinical OCT catheters.

Multimodal images obtained from artery specimen illustrate the complementarity of multimodal approaches, where, e.g., presence of mFC leads to both changes in fluorescence lifetime properties due to ceroids,^12^ and increased attenuation of OCT signal. A joint analysis may lead to improved inflammation quantification. Similarly, improvements in characterization of the lesion’s extracellular matrix may be obtained by combining both spectroscopic and OCT data and will be explored as part of a wider study.

## 5 Conclusion

By integrating key improvements in the areas of catheter design, rotary junction, and FLIm detection, we demonstrated that a reliable intravascular FLIm-OCT system could be designed, without incurring any of the tradeoffs in FLIm or OCT performances evidenced in previously reported FLIm-OCT systems. The ability to perform fast intravascular coregistered time-resolved fluorescence decay measurements in multiple spectral emission bands in combination with OCT is an exciting prospect. Thus, future work will focus on clinical translation of the technology to enable *in vivo* assessment of coronary artery pathology and providing unique insights into the interplay of plaque morphology, composition, and biomechanical properties implicated in plaque progression and mechanisms of thrombosis.

## Code, Data, and Materials availability

Data underlying the results presented in this paper may be obtained from the authors upon reasonable request and through a collaboration agreement.

## References

[1] Virani S. S. et al., “Heart disease and stroke statistics – 2021 update: a report from the American Heart Association,” Circulation 143(8), E254–E743 (2021). CIRCAZ 0009-7322 10.1161/CIR.0000000000000950 33501848 PMC13036842

[2] Bourantas C. V et al., “Hybrid intravascular imaging: recent advances, technical considerations, and current applications in the study of plaque pathophysiology,” Eur. Heart J. 38, 400–412 (2017). EHJODF 0195-668X 10.1093/eurheartj/ehw097 27118197 PMC5837589

[3] Ricciardi C. et al., “Assessing cardiovascular risks from a mid-thigh CT image: a tree-based machine learning approach using radiodensitometric distributions,” Sci. Rep. 10, 2863 (2020). SRCEC3 2045-2322 10.1038/s41598-020-59873-9 32071412 PMC7029006

[4] Kellnberger S. et al., “Intravascular molecular-structural imaging with a miniaturized integrated near-infrared fluorescence and ultrasound catheter,” J. Biophotonics, 14, e202100048 (2021). 10.1002/jbio.202100048 34164943 PMC8492488

[5] Tearney G. J. et al., “Consensus standards for acquisition, measurement, and reporting of intravascular optical coherence tomography studies: a report from the international working group for intravascular optical coherence tomography standardization and validation,” J. Am. Coll. Cardiol. 59(12), 1058–1072 (2012). JACCDI 0735-1097 10.1016/j.jacc.2011.09.079 22421299

[6] Michail M. et al., “Intravascular multimodality imaging: feasibility and role in the evaluation of coronary plaque pathology,” Eur. Hear. J. - Cardiovasc. Imaging 18(6), 613–620 (2017). 10.1093/ehjci/jew330 28329320

[7] Khraishah H. Jaffer F. A., “Intravascular molecular imaging: near-infrared fluorescence as a new frontier,” Front. Cardiovasc. Med. 7(Nov.), 1–13 (2020). 10.3389/fcvm.2020.587100 33330648 PMC7719823

[8] Ma D. et al., “Rotational multispectral fluorescence lifetime imaging and intravascular ultrasound: bimodal system for intravascular applications,” J. Biomed. Opt. 19(6), 066004 (2014). JBOPFO 1083-3668 10.1117/1.JBO.19.6.066004 24898604 PMC4045254

[9] Datta R. et al., “Fluorescence lifetime imaging microscopy: fundamentals and advances in instrumentation, analysis, and applications,” J. Biomed. Opt. 25(7), 071203 (2020). JBOPFO 1083-3668 10.1117/1.JBO.25.7.071203 PMC7219965 32406215

[10] Park J. et al., “Biochemical characterization of atherosclerotic plaques by endogenous multispectral fluorescence lifetime imaging microscopy,” Atherosclerosis 220(2), 394–401 (2012). ATHSBL 0021-9150 10.1016/j.atherosclerosis.2011.10.034 22138141 PMC3264694

[11] Alfonso-Garcia A. Haudenschild A. K. Marcu L., “Label-free fluorescence lifetime imaging for assessment of carotid artery grafts,” Opt. InfoBase Conf. Pap. Part F91-T(9), 1–9 (2018). 10.1364/BOE.9.004064 PMC6157793 30615748

[12] Bec J. et al., Label-Free Visualization and Quantification of Biochemical Markers of Atherosclerotic Plaque Progression Using Intravascular Fluorescence Lifetime, JACC Cardiovasc. Imaging, Elsevier Inc. (2020). 10.1016/j.jcmg.2020.10.004 PMC8116358 33221238

[13] Ughi G. J. et al., “Dual modality intravascular optical coherence tomography (OCT) and near-infrared fluorescence (NIRF) imaging: a fully automated algorithm for the distance-calibration of NIRF signal intensity for quantitative molecular imaging,” Int. J. Cardiovasc. Imaging 31, 259–268 (2015). 10.1007/s10554-014-0556-z 25341407 PMC4344893

[14] Kim A. et al., “Quantification of in vivo fluorescence decoupled from the effects of tissue optical properties using fiber-optic spectroscopy measurements,” J. Biomed. Opt. 15(6), 067006 (2010). JBOPFO 1083-3668 10.1117/1.3523616 21198210 PMC3025598

[15] Sherlock B. E. et al., “Synchronous fluorescence lifetime imaging and optical coherence tomography using a double clad fiber,” in 2016 IEEE Photonics Conf., IPC 2016, Vol. 42, No. 19, pp. 3753–3756 (2017). 10.1109/IPCon.2016.7831145 PMC8951707 28957119

[16] Lee M. W. et al., “Comprehensive intravascular imaging of atherosclerotic plaque in vivo using optical coherence tomography and fluorescence lifetime imaging,” Sci. Rep. 8, 14561 (2018). SRCEC3 2045-2322 10.1038/s41598-018-32951-9 30267024 PMC6162321

[17] Chen X. et al., “Dual-modality optical coherence tomography and frequency-domain fluorescence lifetime imaging microscope system for intravascular imaging,” J. Biomed. Opt. 25(9), 096010 (2020). JBOPFO 1083-3668 10.1117/1.JBO.25.9.096010 PMC7525154 33000570

[18] Bec J. Li C. Marcu L., “Broadband, freeform focusing micro-optics for a side-viewing imaging catheter,” Opt. Lett. 44(20), 4961 (2019). OPLEDP 0146-9592 10.1364/OL.44.004961 31613239 PMC9010228

[19] Bec J. et al., “In vivo label-free structural and biochemical imaging of coronary arteries using an integrated ultrasound and multispectral fluorescence lifetime catheter system,” Sci. Rep. 7, 8960 (2017). SRCEC3 2045-2322 10.1038/s41598-017-08056-0 28827758 PMC5566546

[20] Zhou X. et al., “Multispectral fluorescence lifetime imaging device with a silicon avalanche photodetector,” Opt. Express 29(13), 20105 (2021). OPEXFF 1094-4087 10.1364/OE.425632 34266107 PMC8237936

[21] Kuo W.-C. et al., “Balanced detection for spectral domain optical coherence tomography,” Opt. Express 21(16), 19280 (2013). OPEXFF 1094-4087 10.1364/OE.21.019280 23938845

[22] Liu J. et al., “A novel method for fast and robust estimation of fluorescence decay dynamics using constrained least-squares deconvolution with Laguerre expansion,” Phys. Med. Biol. 57(4), 843 (2012). PHMBA7 0031-9155 10.1088/0031-9155/57/4/843 22290334 PMC3407553

[23] Moon S. Lee S.-W. Chen Z., “Reference spectrum extraction and fixed-pattern noise removal in optical coherence tomography,” Opt. Express 18(24), 24395–24404 (2010). OPEXFF 1094-4087 10.1364/OE.18.024395 21164786 PMC3100290

[24] Kim W. et al., “Lensless, ultra-wideband fiber optic rotary joint for biomedical applications,” Opt. Lett. 41(9), 1973 (2016). OPLEDP 0146-9592 10.1364/ol.41.001973 27128052 PMC6731063

[25] Miller S. W. Wood J. P. Loewenthal S., “Angular runout test setup for high-precision ball bearings,” in 41st Aerospace Mechanisms Symposium, Jet Propulsion Laboratory, pp. 439–450 (2012).

[26] Park H.-C. et al., “Broadband rotary joint for high-speed ultrahigh-resolution endoscopic OCT imaging at 800 nm,” Opt. Lett. 42(23), 4978–4981 (2017). OPLEDP 0146-9592 10.1364/OL.42.004978 29216160 PMC5907933

[27] Hii K. F. et al., “Error motion of a kinematic spindle,” Precis. Eng. 28(2), 204–217 (2004). PREGDL 0141-6359 10.1016/j.precisioneng.2003.11.001

[28] Tearney G. J., “Intravascular optical coherence tomography,” Eur. Heart J. 39(41), 3685–3686 (2018). EHJODF 0195-668X 10.1093/eurheartj/ehy646 30383273

[29] Li J. et al., “Ultrathin monolithic 3D printed optical coherence tomography endoscopy for preclinical and clinical use,” Light Sci. Appl. 9, 124 (2020). 10.1038/s41377-020-00365-w 32704357 PMC7371638

